# On-Site Inspection Form in Veterinary Cases: The Parma Veterinary Form

**DOI:** 10.3390/ani13132064

**Published:** 2023-06-22

**Authors:** Cristina Marchetti, Luigi Mastrogiuseppe, Stefano Vanin, Rossana Cecchi, Mirella Gherardi

**Affiliations:** 1 Independent Researcher, 43100 Parma, Italy; crimar.vet@gmail.com; 2 Department of Prevention, Unit of Veterinary, Regional Health Unit of Molise, ASREM, 86100 Campobasso, Italy; mastrogiuseppe1962@gmail.com; 3 Department of Earth and Environmental Sciences, University of Genoa, 16132 Genoa, Italy; stefano.vanin@unige.it; 4 National Research Council, Institute for the Study of Anthropic Impact and Sustainability in the Marine Environment (CNR-IAS), 16149 Genova, Italy; 5 Department of Medicine and Surgery, Unit of Forensic Pathology, University of Parma, 43124 Parma, Italy; 6 Department of Prevention of the Local Health Authority, SC Medicina Legale AUSL Valle D’Aosta, 11100 Aosta, Italy; mgherardi@ausl.vda.it

**Keywords:** crime scene investigation, on-site inspection form, forensic veterinary sciences, one health, pathology, anatomy, entomology, genetics, toxicology, zoonosis

## Abstract

**Simple Summary:**

In veterinary practice, the on-site inspection of the scene of an animal dead body is crucial for a correct interpretation of the autopsy results, in particular to determine the manner, mechanism and cause of death, as an important role in the control of public health including the prevention of zoonoses. It is also fundamental for the recognition and the contrast of crimes against animals and to animal abuse phenomena, considered an alert sign of an anti-social or violent behavior of humans, theory known as “The Link”. A good practice is the presence of a veterinary pathologist on the scene. Although photographs and information made available by the police officers on the place of discovery of the animal cadaver can be useful, the information that can be achieved by a direct examination of the scene is irreplaceable. Today the best veterinary procedure requires an accurate collection of evidence at the scene that can be then handed to experts belonging to other forensic sciences for further evaluation and data interpretation. In this paper authors suggest a form aiming to facilitate either the on-site and the autopsy activities. The suggested form can contribute to guarantee the quality of the forensic process from the discovery site up to the court. Particular attention is paid to the training of non-medical personnel who often represent the first, and sometimes, the only figure to be present on the scene. The form proposed is inspired by the interdisciplinary form developed by the European Council of Legal Medicine. This form represents an initial tool to improve a multidisciplinary activity in close synergy with other forensic experts.

**Abstract:**

The on-site inspection of the scene of an animal cadaver is crucial for a correct interpretation of the autopsy results, to determine the manner, method, and cause of death. This information plays a crucial role in the control of public health including the prevention of zoonoses. It is also fundamental for the recognition and the contrast of crimes against animals and to animal abuse phenomena, considered an alert sign of an anti-social or violent behavior of humans. Today the best veterinary procedure requires an accurate collection of the evidence at the scene that can be then handed to experts belonging to other forensic disciplines for further evaluation and data interpretation. In this paper authors suggest a form aiming to facilitate either the on-site and the autopsy activities, as a guarantee of the quality of the forensic process starting from the discovery scene up to the reconstruction of the case. Essential is training of non-medical personnel who often represent the first responder to be present on the scene. The form is inspired by the interdisciplinary form developed by the European Council of Legal Medicine and represents an initial tool to stimulate a multidisciplinary activity in close synergy with other forensic experts.

## 1. Introduction

The discovery of an animal cadaver—pet, livestock or wild animal—induces the veterinary pathologist to consider as potential cause of death pathologies or other natural causes. The identification of zoonoses plays also an important role for public health and their identification is a primary responsibility of the veterinary pathologist [1,2,3,4,5,6,7,8,9,10,11,12] who must keep abreast of the monitoring of zoonoses in animals and the environment as part of the One Health approach [13].

During each on-site inspection, the veterinary must ensure the safety of people and more in general the biosecurity of the site. The complexity and coordination of these activities will be adapted to the specific situations by different actions depending on whether it is the death of a single animal or an outbreak of disease or a mass mortality event. 

Veterinary doctors, as well as medical doctors, are acknowledged of infectious and parasitic diseases and the degree of specific and relative risk that contact with infected material (saliva, blood, urine, mucous, feces, or other body fluids of an infected animal) can produce on operators, animals and environment. National and global health organizations have published institutional standard operating protocols, and best practice protocols to be applied in cases of possible sources of zoonotic diseases (e.g., Word Organisation for animal Health Founded as OIE) [14].

As in human forensic pathology, also in veterinary pathology, the on-site inspection is fundamental, such as the autopsy, for the identification of the causes, the manner and the circumstances of the death [15].

Nevertheless, traumatic, unnatural, violent and suspicious causes of death have not to be underestimated and deserve a careful investigation [16]. Any hypothesis on the cause of death must be verified [17,18,19]. In veterinary field it is important, for legal reasons, to differentiate cases of predation from postmortem consumption by wild or domestic animals, both vertebrates and invertebrates [20,21,22]. Therefore, different kinds of evidence (traces, blood pattern, excrement, hairs, bones, etc.), recorded and collected from the discovery site or from the body, may provide useful information [23]. A correct documentation and collection of the evidence is fundamental to satisfy or deny the request of reimbursement, provided by national legislations, when the damage is due to predation by wild animals [24].

This approach is routinely applied when human cases are investigated, but it remains still underestimated and not always applied when animals are involved. In addition, the pressure, the lack of time and the environmental conditions (e.g., wild environment) may cause errors and imprecisions that affect the quality of the investigation. Literature reveals the lack of simple and standardized procedures and recording schemes to be used in veterinary contexts. A tool allowing a standardized and quick collection and documentation of all the available evidence would be beneficial to the veterinary forensic pathologists, thus, reducing the potential loss of information during the scene investigation that affects directedly the accuracy of the reconstruction of the peri- and postmortem events. In addition, the correct conservation of biological samples and the chain of custody have to be guaranteed [25]. The documentation, performed by sketching, photographing and geo-localizing the scene (i.e., using GPS), of a case of veterinary interest is fundamental as in a human case [26,27,28,29]. 

To have a more precise reconstruction of the events, a multidisciplinary approach is required. This approach can be realized only with an effective exchange of information between all the specialists working on the case. 

The presence of a veterinary pathologist on the scene working with the police officers is fundamental but it is achieved only in a very few cases. Indeed, from our experience, in most of the cases the animal cadaver is collected and delivered to the veterinary pathologist often without any information and proper documentation.

The aim of this paper is to present a form to support the veterinary scene investigation activities when a dead or injured animal is found. It also aims to improve the quality of the forensic process, in veterinary contexts, from the scene to the court [30]. Authors believe that a close collaboration between human and veterinary forensic medicine professionals can strengthen awareness of the theory of interspecies violence known as “The Link”. The use of shared tools and a common nomenclature will contribute to this [31,32,33,34,35,36]. The on-site inspection sheets here exposed represent a continuity with the work produced in 2021 by Cecchi and colleagues [37].

## 2. Materials and Methods

This project is inspired by the interdisciplinary on-site inspection form developed by the European Council of Legal Medicine (ECLM): the Parma form [37]. A working group consisting of veterinarians and forensic pathologists belonging to the Parma working group [38] modified the Parma form adapting it to forensic veterinary pathology needs. In general, the definitions of the professions of human medicine have been replaced by those of veterinary medicine. To these can be added other professions still active in the field of first aid such as police and environmental eco/guards. Unlike human forms, veterinary cards have an additional box in which the identification data (microchip and/or ear tag) of the animal victim is entered when the victim belongs to domestic species and, in some cases, to wild species (farmed or released in the wild after capture and application of identification devices). The body patterns of the human have been replaced with animal body patterns of generic species adaptable to all mammals. The same model can easily be adapted to avian, amphibian and reptile species. 

A page has been added to form P in which the pathologist is guided in the collection of evidence from both the environment and the cadaver that is useful in directing the pathologist towards a suspected cause or concomitant cause of death and, above all, takes on the role of a wake-up call against zoonotic hazards. The destination of the cadaver box is another an adaptation of the form to species of veterinary medical interest. The injuries section has a different legend from that of humans. Specifically, the human tab has a symbol indicating gunshot wound (entry and exit). The authors believe that the term circular wound is more appropriate in the veterinary field as the distinctive marks found on human skin are not likewise conclusive in the veterinary field. Osteological cards differ substantially from human cards both in anatomical terms and in their use. They are to be used in the identification of species on the basis of models of mandible proposed. Identification of the age group. is possible by examining skeletal remains according to the proposed scheme.

The on-site inspection veterinary form has been initially tested on forensic and mock (educational) cases in order to verify its applicability and to highlight and amend any potential weakness. A cohort of 83 professionals (7 private veterinarians, 34 students of veterinary medicine, 4 members of the wild rescue center (WRC) and 38 Environmental guards (specialized on animal care) filled the form in real and simulated cases after following an induction pre-practice course. Of these, 47 people worked in more than one case. The feedback provided by this cohort allowed the final editing of the form as presented in this article which the preliminary results of its application of the forms are also provided.

## 3. Results

**Figure 1:**
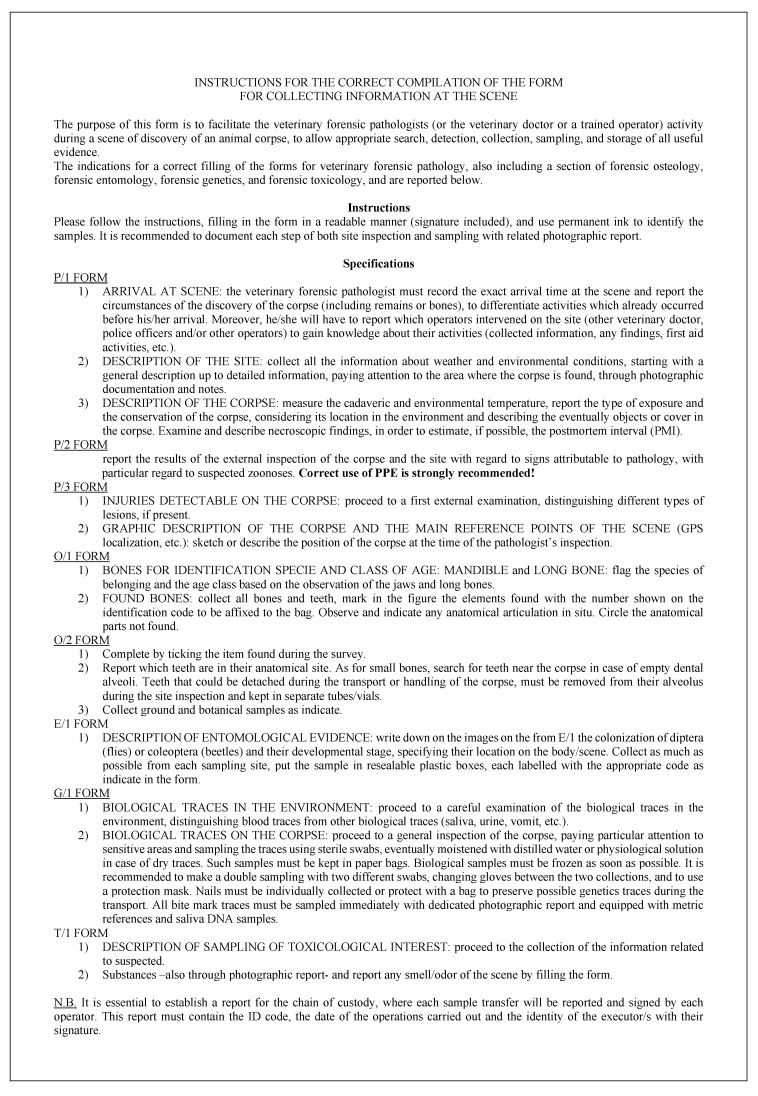

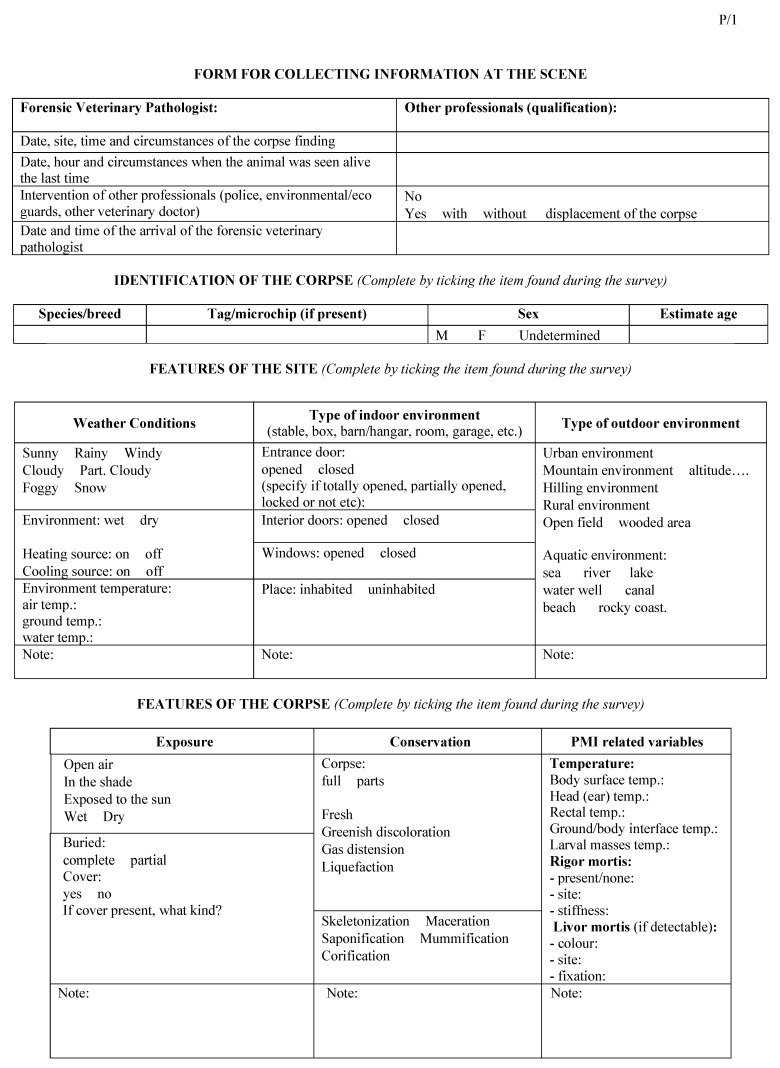

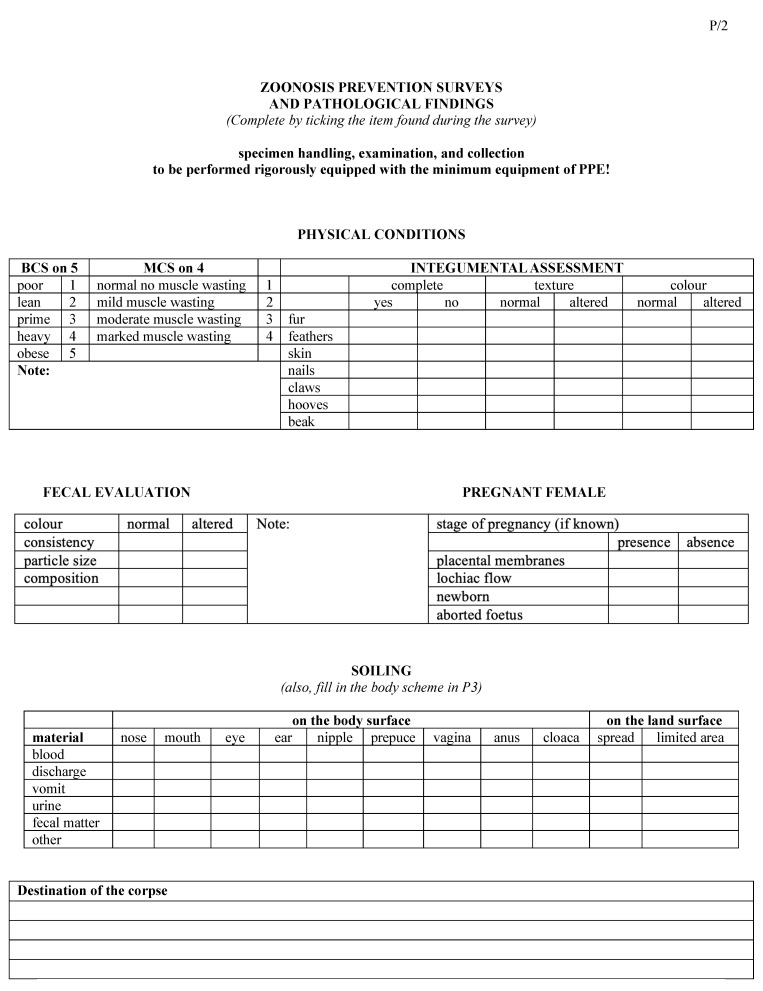

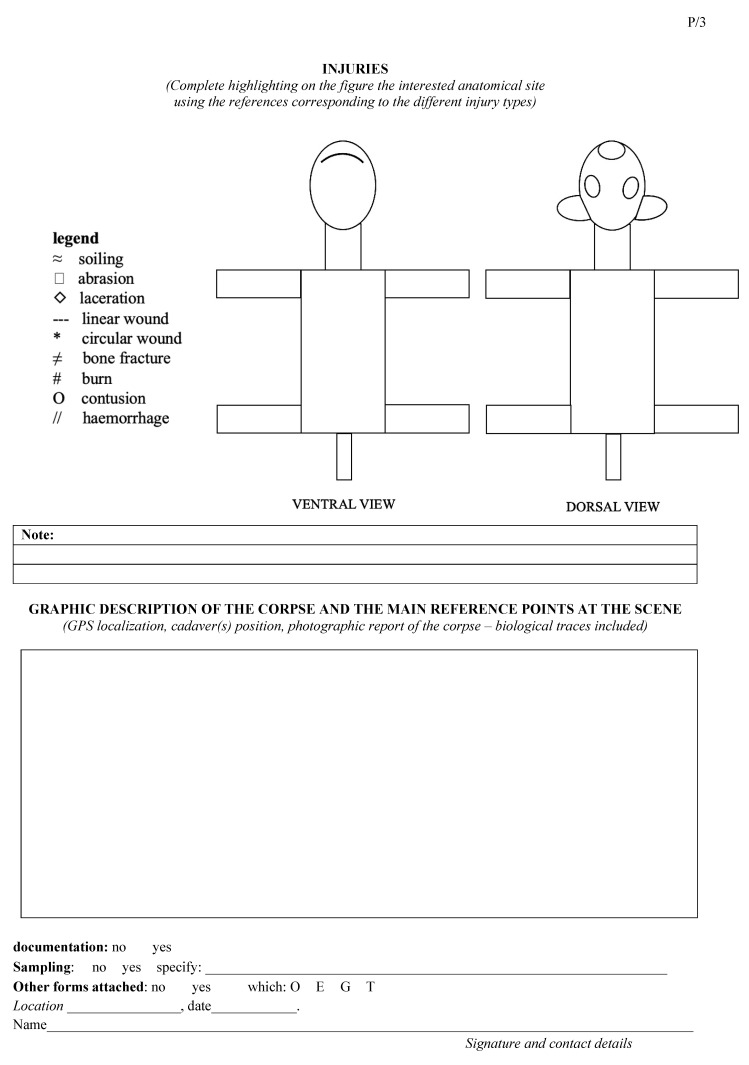

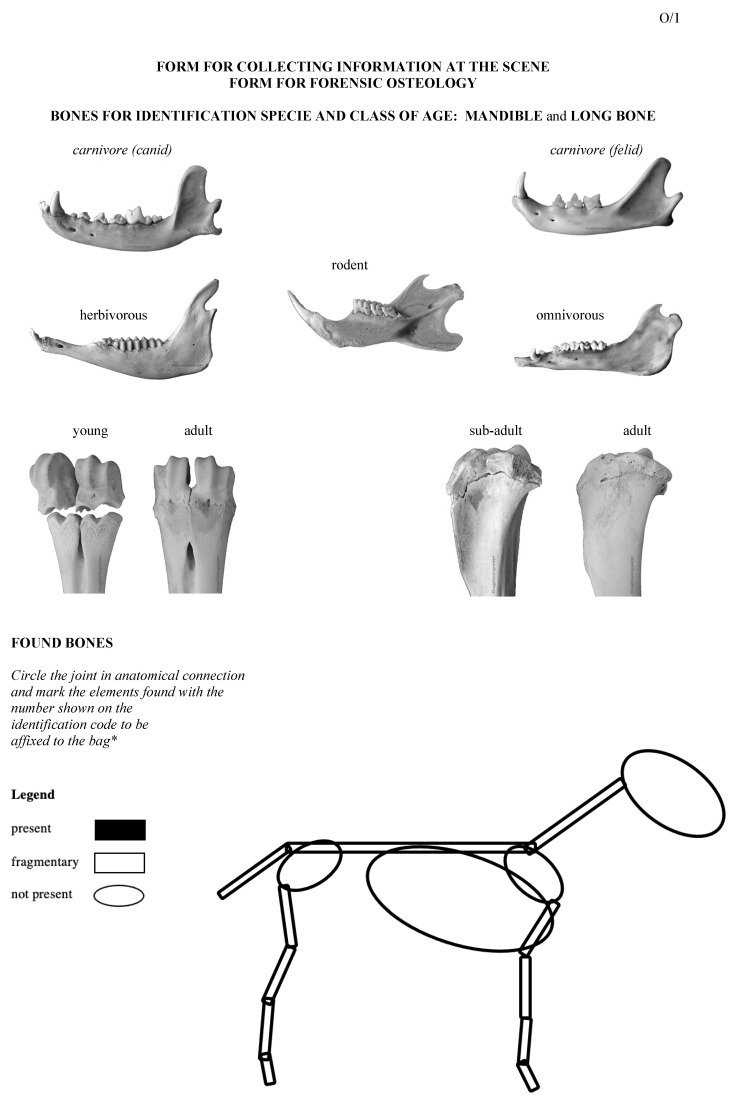

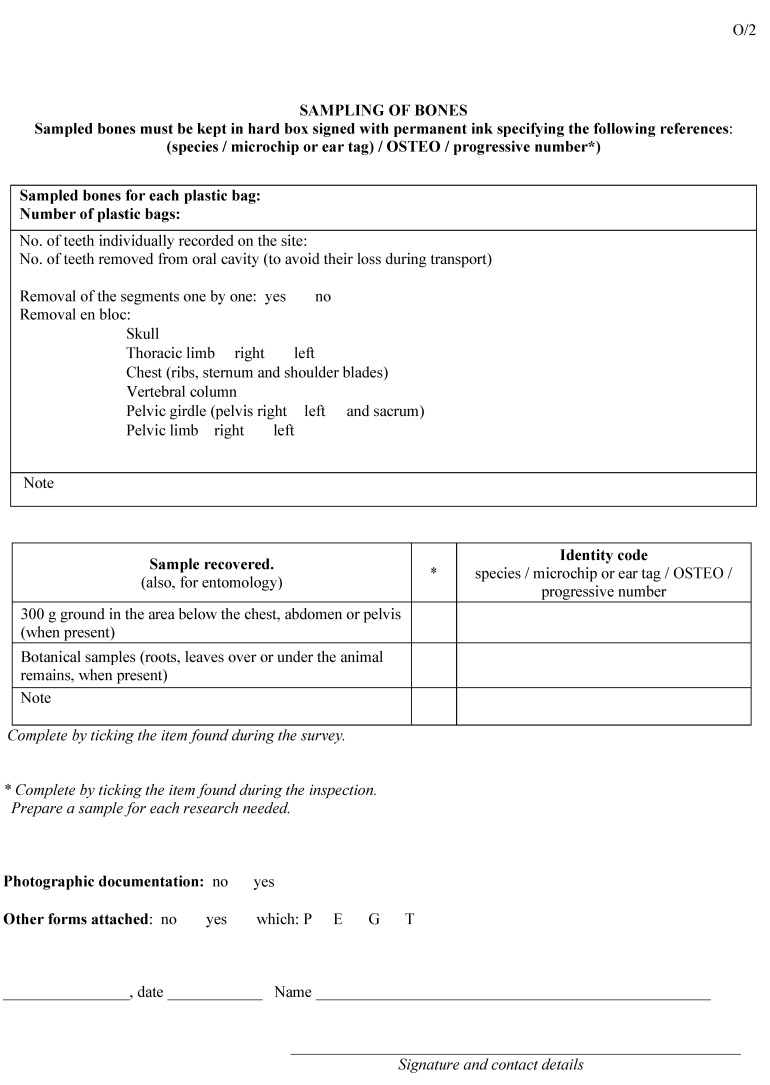

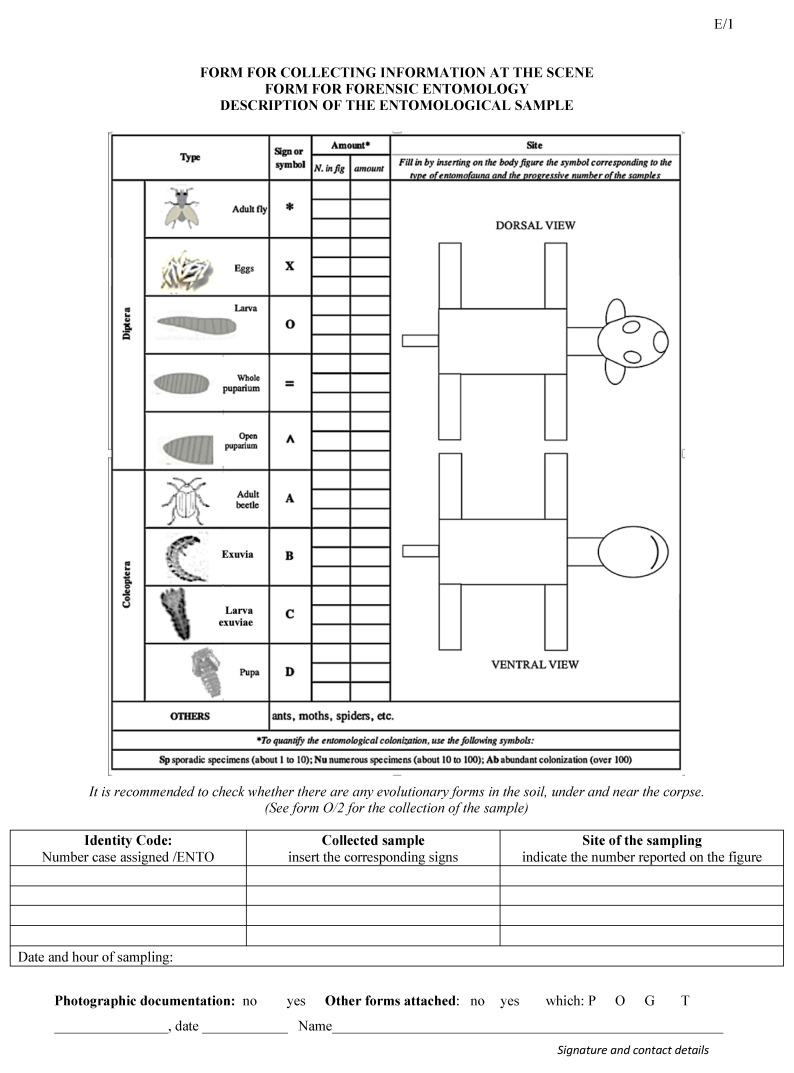

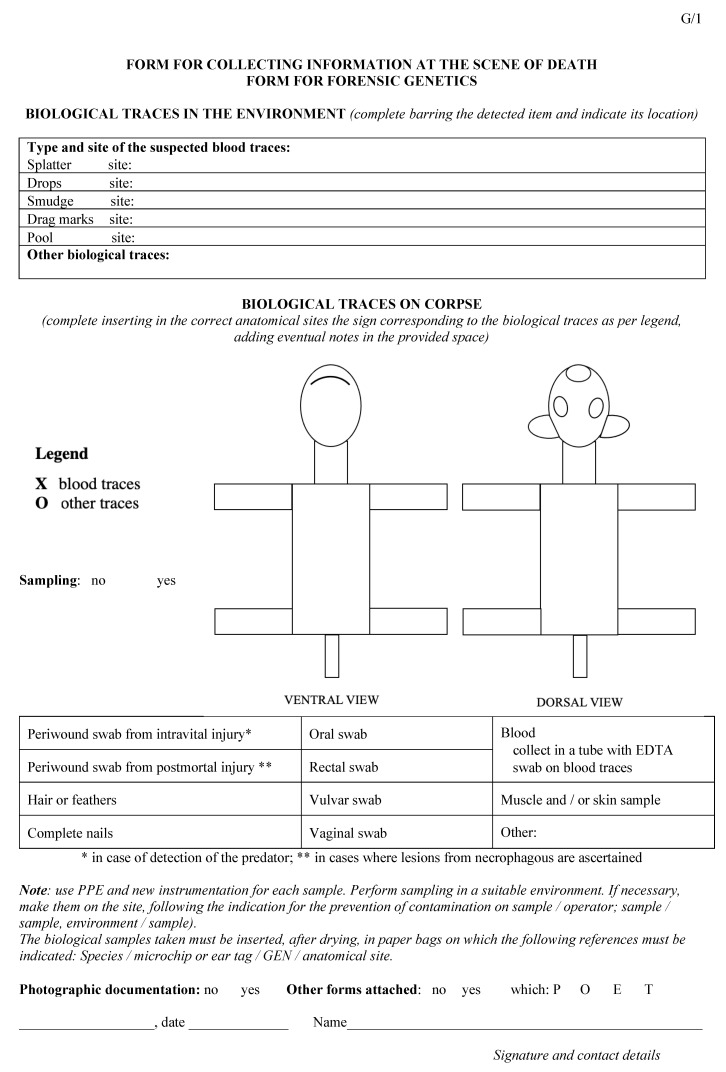

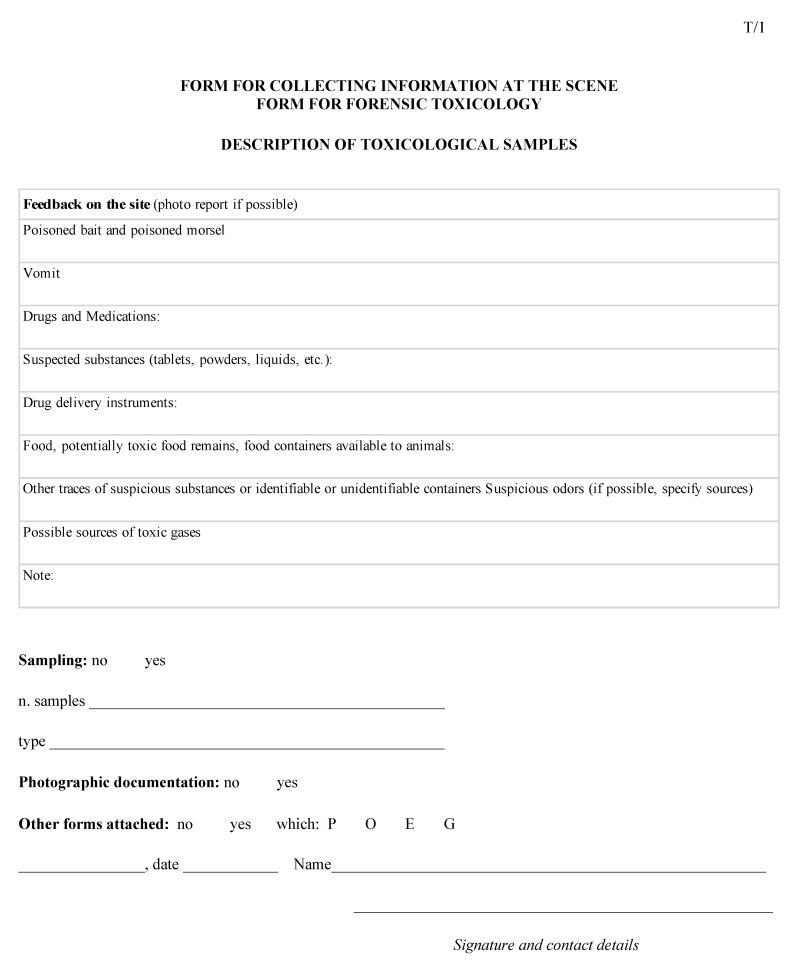
The On-Site Inspection Form in Veterinary Cases: The Parma Form.

Like the Parma form, the on-site inspection veterinary form here proposed, is composed by different independent sheets (Figure 1): three sheets dedicated to veterinary forensic pathology (P/1, P/2 and P/3), two other focused on osteology (O/1 and O/2), one reserved to entomology (E/1), one to genetics (G/1) and one to toxicology (T/1). 

The structure of the cards described below facilitate the veterinary forensic pathologists (or the veterinary doctor or a trained operator) activity during a scene inspection of discovery of an animal dead body, to allow appropriate search, detection, collection, sampling, and storage of all useful evidence. Each sheet reports multiple choices which refer to all the variables that must be observed and have to be specified during the on-site inspection. The operator has only to check the variable of interest and then to fill the relative field/box. Understanding and filling in the card is facilitated and accelerated by body diagrams of a generic mammal and diagrams that are placed at the operator’s disposal. There are also blank frames for additional notes.

Sheet P/1 records the data on the discovery of the cadaver, i.e., date, time and place given as GPS data (GNSS Coordinates). The identity of the person who intervenes at the scene, pathologist, or other professional figure (e.g., the law enforcement authorities), is also reported. The second space is reserved for the identification of the animal. This section shows the species, breed, identification code (e.g., microchip or ear tag) and if any, sex and estimated age. In the following boxes, basic information useful to forensic experts for estimating the time since death and the permanence of the cadaver at the site, are collected. The next box reports the features of the site. In this space, all possible variables relating to the weather conditions are listed and, depending on whether the cadaver was found in an enclosed or an open environment, the operator must flag the entry related to the specific circumstance. In the same way, the last box collects information about the cadaver. The operator can choose between possible options that describe the condition in which the dead animal body is found, for example, if it results covered with objects or partially or totally buried. The operator is then asked to indicate whether the cadaver is present in its entirety or whether only parts are present. With a tick, the operator informs about the state of preservation of the cadaver. Finally, the survey of the listed temperatures is requested, as well as the survey and description of rigor and livor mortis, if present.

Sheet P/2 is devoted to the detection of external signs of the dead body and environment that may lead the veterinary to suspect of an ongoing pathology, information of fundamental importance from a health point of view, regardless of whether this is the cause of death or is a concomitant condition. In particular, the veterinary must report the findings that emerged from the external inspection of the cadaver and of the site with regard for the signs attributable to zoonotic pathology. The correct use of all Personal Protective Equipment (PPE) and the implementation of all procedures necessary to ensure biosecurity for humans, live animals and the environment is strongly recommended. The sheet P/2 helps the veterinary to carefully observe simple details in order to enable him/her to determine the level of biosecurity to be implemented on a case-by-case basis.

The information on soiling already reported in the last section of sheet P/2 is now reported on the generic body diagram. On sheet P/2, soiling consisting of biological material (blood, discharge, vomit, urine, and faecal material) is highlighted while in the sheet P/3 is also requested to specify a soiling from other material such as mud, oily material of different densities, hydrocarbons and so on.

The body diagram on sheet P3 is generic and therefore usable for any terrestrial mammal. On this diagram, the operator marks the location of the soiling or lesions using the symbols listed in the ‘legend’. The last section of P3 sheet reserves an empty space where the location is inserted (GPS localization) also useful for sketch or describe the position of the cadaver at the time of the pathologist’s inspection. The sheet P/3 ends with the annotation of the presence of other forms that must be attached to complete the information intended for each forensic expert.

If skeletal remains are present on the scene, sheets O/1 and O/2 are used. The first part of O/1 is dedicated to the recognition of the species based on mandible and dentition. O/1 sheet is also to be used for and to determine the age range based on ossification by choosing from the images of mandibles and long bones proposed in the form. The operator must flag the recognized jaw and thus indicate whether the bony remains belong to a carnivore (canid or felid), a rodent, an herbivore, or an omnivore. Based on the closing of the physis, the operator chooses the appropriate image between young, sub-adult and adult to indicate the age range of the subject. The second part is used to indicate which bony elements and teeth are present and mark any connections that are still detectable. In O/2, the manner in which the bone and teeth is collected, stored and transported is indicated. Small bones should be searched near the cadaver. In the same way teeth should also be sought if empty dental alveoli are observed. Teeth that may come off and be lost during transport or handling of the cadaver should be removed from their alveolus during site inspection and stored in separate tubes/vials. In the last box, the operator finds the information for the collection of the soil under the cadaver and the botanical elements present around it.

The E/1 sheet is to be used in case of colonization by entomofauna. The sheet shows a column in which are represented the adult forms of Diptera (flies) or Coleoptera (beetles) and their stage of development. The signs and symbols must be pointed out on the mammal generic body scheme located on the right. The operator must collect as much as possible from each sampling site, place the sample in resealable plastic containers, each labelled with the appropriate code, and deliver it to the expert entomologist as soon as possible, following the instructions on the form. Samples will be appropriately set-up for subsequent investigations (e.g., molecular biology methods). There is a space in the sheet where the operator can report the presence of additional insects, which must also be sampled in the same way. 

The G/1 sheet should be used when biological traces are found in the environment or on the dead animal body. For the blood traces on the environment, the sheet offers a list of blood patterns traces from which the operator can choose the most appropriate one. Other biological traces (saliva, urine, vomiting, etc.) should be distinguished and pointed out in the appropriate section. A general inspection of the body is carried out for the collection and storage of biological traces on the cadaver. Particular attention is required for the sampling of areas in which there may be biological elements attributable to the aggressor. Feces or hair must be collected although they are not necessarily indicative of the identity of the aggressor. The module indicates how to sample the traces with sterile swabs, possibly moistened with distilled water or saline in case of dry traces. These samples must be air-dried and stored in paper bags. Biological samples must be frozen as soon as possible. The form is completed with the instructions on the procedure to follow during the sampling that must be carried out with a double sampling with two different pads, changing the gloves between the two collections and keeping a protective mask during throughout the procedure. Special instructions are provided for the nails or claws that must be collected individually or protected with a bag to preserve any genetic traces during transport. All traces of bites inflicted on a live animal must be sampled for the attacker’s salivary DNA. The utility of the sample is linked to the operator’s ability to recognize a bite inflicted on a live animal from the signs resulting from a post mortal consumption. The sample sheet for genetics is supplemented by a body pattern of generic mammalian on which symbols corresponding to blood traces and traces of other organic material found on the cadaver may be inserted. The last part lists the samples that must be considered for sampling. The flag of the samples taken is required.

The T/1 sheet is to be used in case of the presence of substances or materials that lead one to suspect the presence of a toxicant at the scene being examined. The operator is guided in the inspection by a suspect of the presence of substances at the site, including biological material (e.g., vomit), or chemicals in the form of powders or liquids, phyto- and agro-pharmaceutical containers or rodenticide devices, drug containers, but also particular odors. 

In each sheet there is a space to indicate whether photographic documentation was collected, which is strongly recommended.

At the end of each sheet there is a space that must be filled in with name, contact details and date and then signed by the operator.

The completed and signed cards accompany the samples, which must be clearly identified by a unique code affixed on all samples collected during the inspection. Sheets and samples are then transferred along the chain of custody.

Instruction for the correct filling of the form, in short and essential format, is available for the beginners (Figure 1).

**Scheme 1:**
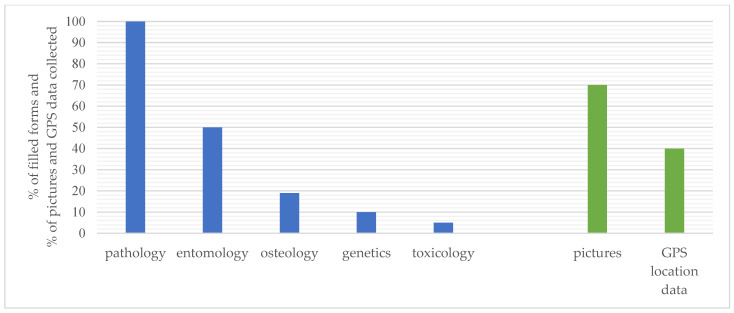
Percentage of filled forms and percentage of pictures and GPS data collected during the on-site inspection (N = 140).

The form was applied to 129 real cases and 11 simulations. In 87 cases the inspection was performed on fresh cadavers and in 20 cases in skeletonized cadavers. Forty-five cases concerned dead animals found on the roadside or in the highway. In 5 cases they were found in urban context (city, garden, etc.). The authors assessed the quality of the compilation of the sheets assessing the completeness and punctuality of completed forms. The GPS location data (GNSS coordinates) has almost always been applied to cases of dead animals found in non-urbanized areas while resulted totally absent in urbanized areas where only the address data has been entered or simply the name of the locality has been indicated. The pathology sheet (P/1, P/2 and P/3) was always completed while the other sheets were compiled only if necessary. In detail, in 100% of the cases the P/1, P/2 and P/3 sheets were filled, in the 50% the E/1 was compiled, in the 19% the O/1, in the 10% the G/1 and in the 5% the T/1. In 70% of the cases pictures were taken (Scheme 1).

## 4. Discussion

Domestic animals, sympatric species and wild animals can be affected by infectious and parasitic diseases. These may represent the cause of death but may also be present without causing death. In any case, it is advisable to consider each cadaver as a potential source of health danger for humans, animals, and the environment. Sheet P/2 includes a space in which the veterinarian must note the destination of the cadaver for the purposes of subsequent diagnostic examinations (autopsy and ancillary examinations), or, if the external examination reveals a suspicion of the presence of transmissible diseases, the veterinarian responsible indicates the specific mode of transport or disposal [39].

Once the site is safe from a health point of view, as well as in human, also in veterinary medicine, well-collected information and samples are fundamental for passive health surveillance in the territory, in particular if the body found belongs to a wild animal.

Today we know that well-collected information and samples are fundamental investigative clues and help to reconstruct cases of abuse and violence against humans and animals.

In the present study, authors believe that collecting and delivering samples to forensic specialists under standardized conditions, i.e., through shared tools, is the key to reconstruct the dynamic of death and clarify forensic issues more quickly and accurately. In order to comply with the chain of custody, the instructions for filling in the forms recall that each sample transfer is correctly identified and signed by each operator.

Authors propose a tool, which shares concepts, procedures and languages common to both pathologists and veterinarians. It is advisable for police officers to be trained in order to fill this tool also when veterinarians are not called on the death scene. The innovative choice of applying a single method to human and veterinary medicine, aims to standardize the presentation of cases of abuse and violence against animals in court. Thus, in order to raise up, through a scientific approach, awareness and availability in the Court to the theory of interspecific violence [40]. The recognition and reporting by veterinarians of a case of violence against animals has a social value of the utmost importance. This is true both in the fight against animal crimes and in the context of One-Health, as it represents a decisive and necessary act to stop the cycle of violence. A close collaboration between the forensic disciplines is the basis of the concept of One-Health in which multiple sectors communicate and work together with a common purpose: the search for truth for the benefit of humans and animals.

The proposed study was tested the on-site inspection form on real and simulated cases highlighted the usefulness of the forms in the standardization of the data collection. The authors’ analysis of the quality of form filling indicates that, in general, the compilers showed adequate knowledge of the subject (e.g., evaluation of the decay of cadavers, skeletal remains, etc.) and ease of use of the tool. At the end of each survey, each participant was asked for a brief discussion regarding their opinion of the tool administered, the identification of difficulties encountered and a judgement on the time taken to complete the form. Each participant appreciated the clarity of the request in the forms, expressed a high degree of satisfaction with the manageability of the tool (only a few sheets), the reliability in the field in not neglecting the collection of elements and the use of the forms as an outline and documentation during the drafting of the report. The feedback of the participants reports that the most difficult part to be filled is the species identification in cases in which the skull or limb extremities were absent, such as in dismemberment due to the scavenger activities. The time taken by the operators to complete the forms and collect the samples was deemed adequate in relation to the degree of exhaustiveness of the surveys conducted.

The negative aspect did not concern the structure of the forms, but some troubles emerged in the acquisition of high-quality images and in the correct collection of biological samples for genetic and entomological analyses. The number of cases in which GPS location data was collected was limited and the information was often inaccurate in its definition. Operators using the proposed on-site inspection forms recognized the value of the location expressed as GNSS coordinates only in rural and wild areas that would not otherwise be identifiable. In urbanized areas, GPS data was never collected but addresses or location names (e.g., village name) were always provided. In one case, the name of a city park was given but no information about the exact spot where the animal dead body was found within the park. To solve these problems, awareness-raising among operators is necessary. In particular, location data using GNSS coordinates should be the object for further training of staff and further study of their importance both for animal and public health and for the study of poaching and animal abuse more broadly. 

The veterinary medical staff, veterinary students, staff employed in the Wildlife Rescue Centers (WRC) and police officers, expresses an overall very positive opinion on the efficacy of the method, both in terms of ease and speed of use, as well as reliability and completeness of the collected data. 

A check and an updating of the forms are advisable according to the continuous evolution of the forensic techniques and the needs of the Court.

## 5. Conclusions

In the present study a quick to use on-site inspection form is presented. It represents a tool to be used by all professionals involved in veterinary forensic field. Using common procedure and language can contribute to an improvement in the forensic discussion of the cases, help in the differential diagnosis between natural and violent death, and supply the Court with scientifically based data.

The concept of One-health plays an important role in the control and prevention of zoonoses by integrating animal, human and environmental health through collaboration and communication between multiple professional sectors, because none of them, individually, can address the problems arising from the interaction animal-man-ecosystem. The authors hope that the correct use of the on-site inspection form proposed here will be applied in all cases of discovery of the cadaver of an animal due to its completeness and ease of application.

## Data Availability

Not applicable.
